# Autonomous or controlled interpreters? Model of *Werktreue* internalization for classical musicians

**DOI:** 10.3389/fpsyg.2024.1401278

**Published:** 2024-07-02

**Authors:** Marie Fujimoto, Yuri Uesaka

**Affiliations:** The Division of Educational Psychology, Graduate School of Education, The University of Tokyo, Tokyo, Japan

**Keywords:** music education, musical interpretation, interpretive autonomy, self-determination theory, self-regulated learning, well-being, performance, the *Werktreue* ideology

## Abstract

Self-regulated learning—a cyclical process in which a learner sets a goal, monitors, and self-reflects on one’s learning to set the next goal—is vital in instrumental learning. However, many conservatory students fail to initiate self-regulated learning; they take lessons passively, practice ineffectively, and fail to give satisfactory performance. These learning experiences could harm students’ well-being, and physical and mental health problems are widespread among students. Nevertheless, factors contributing to self-regulated learning remain unknown. We hypothesized that musicians’ autonomy in musical interpretation, which we refer to as *interpretive autonomy*, plays a pivotal role in self-regulated learning. Without developing interpretation, musicians fail to set personal goals, monitor, and self-evaluate their performances in terms of musicality. Although previous studies imply that interpretation plays a significant role in self-regulated learning, this has not been clearly demonstrated. Studies on interpretive autonomy are scarce due to a complicated discourse surrounding performers’ freedom in interpretation. The ideology of *Werktreue* underpins the classical music field, and classical music performances are evaluated based on how faithfully a performer interpreted the composer’s intention. Yet musicians hold various beliefs regarding the meaning of faithful interpretation, thus the degree of interpretive autonomy cannot be assessed unless its clear definition is provided. In addition, the mechanisms that promote or hinder interpretive autonomy in learning remain unexplained. To address these issues, we proposed a model of *Werktreue* internalization by applying self-determination theory. The model defines interpretive autonomy based on internalization types, identifies its effects on musicians’ learning behavior and well-being, and reveals the mechanisms that promote or hinder interpretive autonomy in learning experiences. This model allows researchers and educators to assess the degree of interpretive autonomy, attribute impaired learning behavior and well-being to a lack of interpretive autonomy, and promote interpretive autonomy by supporting students’ psychological needs in interpretation.

## 1 Introduction

Despite their hard work and dedication, conservatory students face various difficulties in learning. Students tend to be passive in one-to-one lessons, relying on their teachers’ instructions (Gaunt, 2010). In solitary practice, students employ ineffective strategies (Hatfield, 2016; Hatfield and Lemyre, 2016; McPherson et al., 2019), and some students persist in practice even with playing-related injuries (Park et al., 2007). In performance, students become overly self-conscious and give unsatisfactory performances (Clark et al., 2014). These learning experiences endanger learners’ well-being (Perkins et al., 2017), and many classical musicians suffer from both physical and mental health problems (Kenny and Ackermann, 2014).

At other times, students are inspired by teachers in lessons (Gaunt, 2010). In practice, they creatively explore musical ideas (Wise et al., 2017). In performance, when students are fully immersed in the music, they enter a transcendental state, embracing an inherent joy of music-making (Bernard, 2009). These learning experiences promote students’ well-being (Perkins et al., 2017) and personal development (Jääskeläinen, 2023).

Students in the former case lack autonomy, whereas students in the latter case initiate their learning successfully. Student autonomy is considered crucial in instrumental learning (Jørgensen, 2000), and Zimmerman’s (2002) self-regulated learning is a useful framework for investigating student autonomy in learning. Self-regulated learning (SRL) is comprised of three cyclical phases: forethought, performance, and self-reflection (Zimmerman, 2002). In the forethought phase, learners set goals and plan strategies to complete the task and manage motivation for learning. In the performance phase, learners enact learning behavior by employing strategies while monitoring its effectiveness. In the self-reflection phase, learners evaluate their learning performance and attribute failures to appropriate causes. Based on self-reflection, learners then set new goals and plans, initiating the next cycle (Zimmerman, 2002). Researchers show that the framework is adaptable to instrumental learning, and effective SRL is vital for optimal musical development (McPherson and Renwick, 2011; McPherson and Zimmerman, 2011; McPherson et al., 2017).

Nevertheless, what differentiates skillful self-regulators from naïve self-regulators remains unknown. Thus, a lack of self-regulated behavior is often attributed to innate abilities. When conservatory students demonstrated dependent learning behavior, the majority of instrumental professors attributed it to individual traits, such as musical talent and self-confidence (Gaunt, 2008). In addition, support offered by institutions for both teachers and students to reflect on their learning is limited (Jørgensen, 2000; Gaunt, 2008; Burwell et al., 2019). Therefore, it is urgent to identify factors that contribute to SRL to support music students’ learning and well-being.

We argue that autonomy in musical interpretation, which we refer to as *interpretive autonomy*, plays a pivotal role in musicians’ SRL and well-being. Classical musicians interpret meanings from notations on a score, deciding what and how to express a piece. Then, musicians convey their interpretations in performance. Therefore, developing interpretations is a prerequisite for musicians to set a musical goal and plan strategies. Furthermore, interpretation is essential to monitor and evaluate how effectively they conveyed the intended interpretation in performance. In other words, when musicians do not develop interpretation, they cannot make goals, monitor, and reflect on their performances based on musicality, failing to initiate effective SRL. The importance of interpretation in SRL has been implied (e.g., Cantwell and Millard, 1994; Hallam, 2001; Reid, 2001; Hultberg, 2002; Hallam et al., 2012; Hatfield, 2016; McPherson et al., 2019); however, it remains ambiguous. Therefore, although ineffective SRL may be caused by a lack of interpretive autonomy, interpretation has been rarely addressed in both academia and educational practice.

Investigating interpretive autonomy is challenging due to a complicated discourse surrounding musicians’ freedom in interpretation. Musical interpretation is an ill-defined problem; there is no definite “right” solution or answer in interpreting a piece. This allows classical musicians to cultivate their creativity in forming and expressing an individualized interpretation of a piece (Payne, 2016; Wise et al., 2017; Héroux, 2018). However, not all interpretations are accepted in the classical music field. There is a norm that has been regulating performance practice since the end of the 18th century: the ideology of *Werktreue* (Goehr, 1992). Under the norm of being true to the work, musicians’ interpretations are assessed based on their faithfulness in realizing the work the composer intended (Goehr, 1992). With the establishment of “authentic” interpretations in the 20th century, interpretations deviating from normative interpretations, performance styles, or scores often faced disapproval, being deemed disrespectful to the composer and work (Taruskin, 1995). Critics claimed that musicians reproduced normative interpretations to be regarded as faithful interpreters, and this led classical music performances to be static and monotonous (Szigeti, 1979; Small, 1986; Taruskin, 1995; Adorno, 2006; Leech-Wilkinson, 2020).

Although a lack of interpretive autonomy among professional musicians and student musicians has been pointed out (Szigeti, 1979; Small, 1986; Taruskin, 1995; Hultberg, 2002; Adorno, 2006; Leech-Wilkinson, 2020), assessing the degree of interpretive autonomy is challenging due to varying definitions of interpretation. Musicians internalize the *Werktreue* ideal differently and hold various concepts of faithful interpretation. Silverman (2007) organized them into two contrasting views: formalist and subjective. Musicians with the formalist view aim to “[let] the score speak for itself” by strictly adhering to scores (p. 102). In contrast, musicians with the subjective view interpret based on their subjective feelings and desires. Silverman argued that musicians with the formalist view fail to convey personal interpretations in performance, as they perceive themselves as “merely the ‘servant’ of the composer” (p. 102). However, musicians holding the formalist view cannot be automatically considered to lack autonomy or individuality in their interpretations. These musicians may genuinely wish to adhere to the score to be faithful to the composer’s intention, and every musician has the right to embrace their principles in interpretation. In addition, when musicians make faithful interpretive decisions, they are ultimately expressing personal musical choices, as composers’ intentions are unknowable (Cook, 2013).

In addition, mechanisms that promote or hinder interpretive autonomy in learning remain unknown. Previous studies have suggested that open questions and dialogue on musical characters are effective in promoting students’ interpretations at the pre-college (Meissner, 2017; Meissner and Timmers, 2019, 2020; Meissner et al., 2021) and college levels (Hultberg, 2002; Young et al., 2003; Burwell, 2005; Nerland, 2007). Conversely, authoritarian teaching that rejects students’ musical ideas has been cautioned to impede the development of artistic voice (Persson, 1996; Silverman, 2008; Wagner, 2015). Nonetheless, the underlying mechanisms of the promoting or suppressing effects of these teaching methods on interpretive autonomy remain unexplained.

Factors that nurture or impede interpretive autonomy in learning experiences must be identified to ensure that a lack of interpretive autonomy is not mistaken for a lack of talent; as with SRL, the degree of interpretive autonomy is easily attributed to an innate ability. Some conservatory students were suspicious that interpretation can be taught or learned (Burwell, 2014). Attribution of innate ability is also observed in expressivity (Lindström et al., 2003; Laukka, 2004) and musicality (Kingsbury, 1988), which are both closely related to interpretation. However, attribution to innate ability can make learners vulnerable to negative feedback (Dweck, 1999). For instance, when a conservatory student received feedback from professors that she was “unmusical” in an exam, her self-esteem was negatively impacted because she regarded unmusicality as unchangeable (Kingsbury, 1988, p. 65).

To summarize, three research gaps exist in interpretive autonomy. First, consensus on the definition of interpretive autonomy is lacking, hindering research on interpretive autonomy. Second, the mechanisms that promote or hinder interpretive autonomy are unknown, allowing the degree of interpretive autonomy to be attributed to innate ability. Finally, the significance of interpretive autonomy in musicians’ learning is implied but remains ambiguous. Because of these gaps, students’ autonomy in interpretation is overlooked in both academia and educational practice. To address these issues, we propose a theoretical model that defines interpretive autonomy, explains the mechanisms that promote or hinder interpretive autonomy in learning, and identifies its effects on musicians’ learning and well-being. Following self-determination theory (Ryan and Deci, 2000, 2017), we define interpretive autonomy based on types of internalization of the *Werktreue* ideal: autonomous and controlled. The model offers a new rationale for educators and researchers to recognize the importance of interpretive autonomy and consider factors affecting interpretive autonomy in music education.

The article is organized as follows. First, we examine maladaptive learning behavior found in lessons, practice, and performance, and we investigate the role of interpretive autonomy in effective SRL. In addition, we address the importance of SRL for musicians’ well-being. Second, we briefly overview the historical background of the *Werktreue* ideology, illustrate its impact on performance practice, and address criticisms against “authentic” performers. Third, we introduce varied interpretive approaches adopted by musicians and categorize them into approaches that indicate interpretive autonomy or lack thereof. Finally, we present the model of *Werktreue* internalization and discuss its implications for educators and researchers in music education.

## 2 Interpretive autonomy, learning behavior, and well-being

This section discusses the relationship between interpretive autonomy and learning behavior in lessons, practice, and performance. SRL has been applied to investigate musicians’ learning in practice (see Varela et al., 2016 for a systematic review) and performance (Williamon et al., 2017). Extending the application into the context of lessons, we intend to comprehensively capture student learning behavior in the three contexts: lessons, practice, and performance^1^.

### 2.1 Other-regulated learning in lessons

Historically, instrumental learning has taken place in one-to-one tuition where masters demonstrate and students imitate (Jørgensen, 2000; Nielsen, 2006; Burwell, 2013). Teachers are experienced professional practitioners whose knowledge and skills reflect norms and expectations in the professional community of the field (Nerland, 2007). As students acquire musical knowledge and skills, they also acquire competencies and identity as professional classical musicians (Nielsen, 2006; Burwell, 2013).

Although the master-apprenticeship has been proven to be effective in transmitting expertise, researchers warn that student autonomy can be neglected or even impaired in the relationship (Persson, 1996; Jørgensen, 2000; Rostvall and West, 2003; Young et al., 2003; Burwell, 2005, 2021; Karlsson and Juslin, 2008; Silverman, 2008; Gaunt, 2011; Carey and Grant, 2015; Wagner, 2015; Burwell, 2021). Gaunt (2010) interviewed 20 conservatory students and found that students failed to initiate cycles of planning, monitoring, and self-evaluating in lessons. Students did not have long-term goals related to personal development and failed to communicate concerns during lessons. After the lessons, they blindly followed instructions without evaluating them critically. In addition, students in this study were overtly self-critical, possibly caused by their lack of initiative in lessons (Gaunt, 2010).

#### 2.1.1 Interpretive autonomy and self-regulated learning in lessons

Studies suggest that interpretive autonomy helps students initiate SRL in lessons. Reid (2001) categorized students’ understanding of learning into five categories and explored how it related to student learning behavior. At the first two lowest levels, students did not consider interpretation part of musical learning and aimed for the correct technical execution of the music. These students relied on their teachers’ technical instructions to play a piece. In contrast, students who included developing interpretation in their learning goals were “able to make judgments about the appropriateness of their teacher’s advice for their own musical situation” (p. 32). They integrated a teacher’s ideas with their own and explored ideas outside the lessons. At the highest level in the classification, students aimed to find personal meaning and express themselves by conveying their original interpretations to audiences. These students regarded teachers as facilitators for their personal development. This shows that having interpretive autonomy allows students to critically evaluate and adopt their teachers’ instructions for their artistic development, initiating SRL in lessons.

### 2.2 Other-regulated learning in practice

Students often practice in isolation. Instrumental teachers rarely address practice methods in lessons (Jørgensen, 2000), and naïve learners tend to employ ineffective practice strategies, such as playing through a piece while ignoring mistakes (Pitts et al., 2000; McPherson and Renwick, 2001; Hallam et al., 2012). Even in higher education, students practice ineffectively without setting goals, and they fail to concentrate in practice (Hatfield, 2016; Hatfield and Lemyre, 2016). In addition, students may suffer from playing-related injuries, such as all six students in Hatfield’s (2016) study. This implies that ineffective practice results in excessive workload and may cause injuries, as the amount of practice time is correlated with playing-related injuries (Robitaille et al., 2018; Gembris et al., 2020; Macdonald et al., 2022). However, even with the injuries, students and professional musicians tend to play through the pain; their identity is deeply entrenched with music, and quitting performance may result in an identity crisis (Park et al., 2007; de Kock et al., 2023).

#### 2.2.1 Interpretive autonomy and self-regulated learning in practice

Interpretive autonomy helps students initiate SRL effectively in practice. First, active development of interpretation allows students to understand scores deeply. Cantwell and Millard (1994) investigated how students’ “surface” and “deep” learning approaches are related to their levels of understanding of scores. They gave three new scores to six 14-year-old students and interviewed them to understand how they would learn the pieces. They found that a surface approach was characterized by a lack of consideration for interpretation. Students with a surface approach perceived the scores as well-structured problems that could be solved technically. They aimed at achieving literal accuracy of the scores, failing to connect the notations with musical concepts. In contrast, a deep approach was characterized by the active development of interpretation. Students with a deep approach perceived the scores as ill-structured problems of musicality, translating notations into musical themes, expressions, and characters. This difference was found among two pairs of students who held the same grades given by the Australian Music Examinations Board; thus, Cantwell and Millard (1994) concluded that consideration of interpretation differentiated how deeply students processed the scores.

Moreover, interpretive autonomy allows students to employ various practice strategies effectively. Reid (2001) demonstrated that students who did not consider interpretation as part of instrumental learning were limited in their exploration of musical ideas, whereas students who aimed to develop interpretation experimented with different phrasings of musical material. Other studies have shown that, to develop interpretations, students employ diverse strategies that do not necessarily involve playing, such as listening to recordings, singing, performing score analysis, conducting, or creating narratives (Cantwell and Millard, 1994; Reid, 2001; Volioti and Williamon, 2017, 2021; Wise et al., 2017; McPherson et al., 2019). On the other hand, a questionnaire survey among 3,325 children aged 6–19 years indicated that the use of ineffective practice strategies, such as merely running through a piece, is negatively correlated with development of interpretation (Hallam et al., 2012). These studies support that development of interpretation is vital in employing effective practice strategies.

In addition, interpretive autonomy contributes to efficient technical improvement. In the absence of interpretation, students practice techniques without a musical goal. Consequently, these students rely on the quantity of practice to evaluate the effectiveness of practice, such as students in Reid’s (2001) study. In contrast, students who develop interpretation hone techniques to convey a musical expression they intend to convey (Reid, 2001; Wise et al., 2017). McPherson et al. (2019) compared the practice behavior of two first-year university students—one student in the top 5% of their cohort, and the other in the bottom 5%—and found that the high-scoring student aimed to develop personal interpretation and practiced an étude with a goal to improve a specific finger movement. By contrast, the low-scoring student did not have any aims relating to interpretation and played an étude from the beginning “because [she] did not know what [she] was going to do” (p. 27).

A deeper understanding of a score, employment of diverse practice strategies, and efficient technical improvement can lead students to experience inherent enjoyment of learning music. In the above-mentioned study by McPherson et al. (2019), the high-achieving student had an intrinsic motivation for practice and was satisfied after practice with a sense of accomplishment. The latter student practiced how she “normally practice[d], just doing what the teacher says, hopefully” (p. 28), and she left practice with a sense of guilt and helplessness; yet she did not “know what to do next” (p. 29). This indicates that active development of interpretive ideas contributes to effective SRL which leads students to experience inherent satisfaction from practice.

### 2.3 Other-regulated learning in performance

Conservatory students often perform in highly stress-inducing settings, such as exams, auditions, and competitions. In these contexts, the audiences comprise expert musicians, and students are aware that the audiences’ assessment may impact their careers (Perkins, 2010; McCormick, 2015). In addition, performance opportunities are infrequent and unevenly distributed, which imposes pressure on students to obtain high evaluations from the expert audience (Perkins, 2013; McCormick, 2015). Accordingly, 58% of 80 German music students aged 15–19 answered that the status of the audience strongly affected their level of performance anxiety. For these students, performance situations where their teachers and professors were in the audience triggered the highest levels of music performance anxiety (Fehm and Schmidt, 2006).

Self-reflection after performance is also challenging. Daniel (2001) found that the majority of students at an Australian conservatory showed dependence on teachers’ feedback to assess their own performance. However, feedback from others is often vague (Juslin and Laukka, 2000) and contradictory even among experts (McCormick, 2015; Wagner, 2015). Nonetheless, conservatory students take feedback from experts personally, making themselves vulnerable to negative feedback (Kingsbury, 1988; McCormick, 2015). Daniel (2001) reported that when students watched videos of their performances for the first time in the class for self-reflection, 43% made criticisms, such as “I hated it” (p. 222). This implied that the students were overtly self-critical and had difficulties in self-assessment of their performance (Daniel, 2001). This is a serious problem, as students cannot learn from performance experience or set a mastery goal for the next performance without constructive self-reflection.

#### 2.3.1 Interpretive autonomy and self-regulated learning in performance

Even in this challenging situation, interpretive autonomy helps musicians self-regulate themselves in performance. First, having interpretation helps musicians set a musical goal. Clark et al. (2014) investigated musicians’ thoughts before, during, and after a performance, and they found that a musician who gave a successful performance was “absolutely 200% sure of what [the musician] was doing, musically,” whereas another musician who gave a less successful performance admitted that their focus was on technical difficulties of the piece and “nothing on the character” (pp. 26, 28).

Several intervention studies have indicated that focus on musical interpretation facilitates musicians’ performance. Hatfield (2016) conducted a 15-week intervention to promote SRL where he encouraged students to focus on musical expression that they wanted to convey before performance. This helped the students concentrate on music, and they experienced greater satisfaction in performance. Chen (2023) implemented a six-month experiment in which 150 conservatory students took classes specifically on musical interpretation to investigate whether a focus on interpretation promotes the experience of flow in performance. In classes, the students were exposed to different genres of music, and they analyzed and arranged classical music pieces as well as composed their own pieces. After the intervention, the ratio of students who reported a high experience of flow in performance increased 10% from 66.7 to 76.7% in the experimental group. In the control group, in which students experienced traditional conservatory training, the ratio increased only 0.9% from 68.3 to 69.2%.^2^ These findings support that interpretive autonomy improves performance.

### 2.4 Ill-being

Due to challenging conditions—acquiring and maintaining complex performance techniques and being constantly exposed to public scrutiny—many musicians experience physical and mental health problems (Kenny and Ackermann, 2014). Students and professional musicians commonly suffer from performance-related musculoskeletal pain (Fishbein et al., 1988; Ginsborg et al., 2009; Steinmetz et al., 2012; Kenny and Ackermann, 2015; Robitaille et al., 2015; Macdonald et al., 2022). In addition, musicians’ mental wellness is endangered. Questionnaire surveys revealed that music students reported a higher level of anxiety and depression than non-music students in Germany (Spahn et al., 2004) and Norway (Vaag et al., 2021). Music performance anxiety is commonly experienced (Barros et al., 2022), and to ease anxiety, both students (Hernández et al., 2018; Lupiáñez et al., 2022) and professional musicians (Fishbein et al., 1988; Kenny et al., 2014) have reported substance use, including prescribed and non-prescribed medication, alcohol, and illicit drugs.

#### 2.4.1 Self-regulated learning and well-being

Other-regulated learning behavior may increase the risks of musicians’ physical and psychological health problems. Students perceive issues like conflict with teachers, excessive practice, and stressful performance experiences as harmful to their well-being (Perkins et al., 2017). Conversely, support from others, a sense of personal growth, and enjoyment in performance were perceived as beneficial for their well-being (Perkins et al., 2017). This implies that initiating SRL in lessons, practice, and performance is essential for musicians’ well-being, particularly since they are constantly exposed to pressures owing to the nature of the profession.

### 2.5 Summary

This section reviewed the relationship between interpretive autonomy and SRL. In addition, we showed that SRL is essential for not only musical development but also optimal well-being. We now turn to the norm in the classical music field: the ideology of *Werktreue*. The next section presents how powerfully the norm regulates musical interpretation. Furthermore, we address criticisms directed at “authentic” performers that their interpretations lacked individuality. The overview is drawn from musicological literature, primally based on Goehr’s (1992) *The Imaginary Museum of Musical Works: An Essay in the Philosophy of Music.*

## 3 The ideology of *Werktreue*

### 3.1 In the 19th century

For classical musicians to be true to the work, music needs to be considered as a work. However, before 1800 music was not yet recognized as an artistic work but a function to serve in a church and at court (Goehr, 1992). Composers composed music for social events and dedicated their compositions to their employers. Scores were left incomplete, and composers often performed or conducted their own compositions differently each time. Improvisation was also a common practice as a form of public entertainment. In short, music was played for social occasions, and the distinction between composition and performance was blurry (Goehr, 1992).

During the early 19th century, a paradigm shift occurred that drastically changed the ways composers, audiences, and performers engaged with music; the work-concept emerged, in which music was regarded as a *work*. Composers began to identify themselves as freelance artists and asserted their compositions as creative works that had artistic and monetary value independent of performances (Goehr, 1992). Recitals were invented, in which audiences learned to listen to musical works for their own sake. Past compositions by Bach and Mozart were introduced as “timeless masterpieces” that “gave to early composers and their music what they had never had in their lifetimes—precise notations, multiple performances, and eternal fame” (Goehr, 1992, p. 247).

The emergence of the work-concept gave birth to the *Werktreue* ideology, which significantly influenced musicians’ approach to interpretation. Composers provided complete scores to represent their imaginary work, and performers became responsible for realizing works faithfully by interpreting composers’ true intentions from scores.

“Performers should interpret works in order to present the work as it truly is with regard to both its structural and expressive aspects. Room was to be left for multiple interpretations, but not so much room that interpretation would or could ever be freed of its obligation to disclose the real meaning of the work. A performance met the *Werktreue* ideal most satisfactorily, it was finally decided, when it achieved complete transparency. For transparency allowed the work to ‘shine’ through and be heard in and for itself” (Goehr, 1992, p. 232).

However, the *Werktreue* ideology did not suppress musicians’ individuality in interpretation nor limit interpretive possibilities to written scores in the 19th century. Performers’ unique imagination, inspiration, and creativity were recognized as essential to bring compositions alive (Hunter, 2005). Improvisation remained part of performances in regular concerts, and performers added notes to pre-composed pieces or manipulated tempo freely (Hamilton, 2008). When Liszt performed Fugue by Handel in 1840, Liszt was praised in *The Times* for his performance with “scarcely any additions, except a multitude of ingeniously contrived and appropriate harmonies, casting a glow of color over the beauties of the composition, and infusing into it a spirit which from no other hand it ever before received” (Williams, 1990, p. 135).

#### 3.1.1 The criticisms against authentic musicians in the 19th century

Interestingly, with the emergence of the *Werktreue* ideal, criticisms started to appear that musicians lacked expressivity in the name of authenticity. In his lectures on aesthetics, Hegel cautioned not to “sink to being merely mechanical” in faithful reproduction of works (Hegel, 1975, p. 956).

“The executant has a duty to give life and soul to the work in the same sense as the composer did, and not to give the impression of being a musical automaton who recites a mere lesson and repeats mechanically what has been dictated to him” (Hegel, 1975, p. 956).

Leistra-Jones (2013) argued that Brahms and Joachim intentionally presented themselves as authentic performers by demonstrating extremely serious attitudes toward performance. This self-restraint style was criticized by Wagner in 1869 in his essay *Über das Dirigieren* as “wooden” which “degraded the works they purported to serve” (Leistra-Jones, 2013, p. 420). Wagner considered their performance style was caused by “fear, repression, and conformity rather than idealistic self-denial,” and he regarded their authenticity as “actually a way of concealing a fundamental inability to ‘feel’ music” (pp. 420–421).

### 3.2 In the 20th century

In the 20^th^ century, the *Werktreue* ideology started to impose more restrictions on musicians. From the 1920s, objectivity started to be emphasized over performers’ subjectivity in interpretation (Stenzl and Zedlacher, 1995). Performers sought faithful interpretations based on musical structures and historical documents (Taruskin, 1995). When Stravinsky composed *Octet for Wind Instruments* in 1923, he requested performers to focus on execution instead of interpretation, arguing that his work would be distorted by performers’ interpretation (White, 1985). International competitions were founded, in which faithful performers were awarded as prominent young stars (McCormick, 2015). Recordings became popular and were marketed for their authentic renditions of a piece (Taruskin, 1995). The developments in musicology, competitions, and recordings contributed to establishing the “authentic” interpretation of each piece, which was then shared worldwide.

#### 3.2.1 The criticisms against authentic musicians in the 20th century

Toward the end of the 20th century, criticisms against classical musicians intensified. Critics claimed that musicians lacked individuality in interpretation, thereby monotonizing performances. Under the enforced *Werktreue* ideal, musicians restricted interpretive choices to musical notations (Adorno, 2006), historical facts (Taruskin, 1995), and performance styles (Leech-Wilkinson, 1984). Small (1986) detested concerts where players mechanically reproduced standardized interpretations, and audiences became “skilled at detecting deviations from the written text, either deliberate or accidental, and such deviations incur their severe disapproval” (p. 14). Szigeti (1979) and Adorno (2006) complained that young musicians strived for career advancement rather than artistic development, and musicians assimilated into normative interpretations. Such young musicians’ “second-hand interpretation, accomplished through imitation [of the recordings], is bound to lack the conviction of a personalized conception” (Adorno, 2006, p. 24) and “the stamp of authenticity” (Szigeti, 1979, p. 18).

Taruskin (1995) argued that the cause of self-imposed restrictions in interpretation was deeply ingrained in musicians’ psychology. He mocked the absence of autonomy in musicians as “a failure of nerve, not to say an infantile dependency” (p. 98).

“‘Responsible performers’ are a single type—the modernist type, the type with the punitive *Werktreulich* superego, the type eager to be controlled by the composer and by the composer’s surrogates both animate and inanimate, the type Stravinsky liked and the New Grove approves, who does not ‘interpret’ but ‘transmits.’ *…* Such performers are more likely than any others to repress the manifold authenticated historical practices that demand creative departures from the text. … They certainly have no lock on authenticity” (Taruskin, 1995, pp. 46-47).

These critics did not advocate for abandoning the ideology. They were criticizing performers who strictly obeyed notations, relied on historical facts, or imitated others’ interpretations out of insecurity, willing to conform rather than pursue individuality in interpretation.

### 3.3 In the 21st century

In the 21st century, the *Werktreue* ideology has continued to regulate musicians’ interpretation. Hunter and Broad (2017) identified three characteristics of the ideology in the current classical music world: “to do justice to (or ‘respect’) ‘the composer’s intentions,’” avoidance of “overt intrusion of ‘ego’ in performance and interpretation,” and the score as “the ultimate arbiter of interpretative limits” (pp. 255–257). They investigated conservatory students’ views on the *Werktreue* ideology and found that students were struggling to develop original interpretations within the unclear border of authenticity. Students were aware that they were left with several interpretive choices, but they also recognized that making a wrong choice would result in rejections from gatekeepers.

Some musicologists continue to argue for the reconsideration of musicians’ attitudes toward the ideology (Silverman, 2007; Cook, 2013; Doğantan-Dack, 2017; Leech-Wilkinson, 2020). However, studies on the *Werktreue* ideal are scarce, implying the ideology is taken for granted by both musicians and researchers.

### 3.4 Summary

This section reviewed how the ideology of *Werktreue* has underpinned the classical music field since the 19th century. In the 20th century, authentic interpretations were established and became a benchmark for evaluating performances. Musicologists noted that musicians were anxious about their own musical decisions, lacking autonomy in interpretation. They argued that this led performances to be monotonous, but our review in section 2 has demonstrated that a lack of interpretive autonomy also negatively affects musicians themselves, as it impedes SRL and well-being. The next section reviews music psychological studies to explore how classical musicians approach interpretation, and how their approaches are affected by the *Werkreue* ideal. Moreover, we introduce our categorization of interpretive approaches based on an indication of interpretive autonomy or lack thereof.

## 4 Classical musicians’ approaches to interpretation

Rather than musicians’ beliefs regarding how the *Werktreue* ideal should be, we focused on musicians’ behavior—how musicians approach interpretation. Psychological studies show that conservatory students, teachers, and professionals take different interpretive approaches (e.g., Hallam, 1995; Héroux, 2016, 2018). We examined six contrasting pairs of interpretive approaches; one of each pair reflects a lack of interpretive autonomy and is related to other-regulated learning, whereas the other implies interpretive autonomy and is related to SRL. Furthermore, we demonstrate how the approaches that imply a lack of interpretive autonomy were derived from the *Werktreue* ideal.

### 4.1 Impersonal vs. personal

Héroux (2018) investigated the process of developing interpretation by observing how nine professional guitarists learned a new modern piece. In her study, six musicians used extra-musical elements, such as creating their own stories and recalling personal memories. However, this personal approach was considered inappropriate by another musician, as it made him think that “he had placed himself above the piece,” disrespecting the composer (Héroux, 2016, p. 320). This caused him to refrain from creating narratives and conduct score analysis instead. Such impersonal approach deviated from the ideology of *Werktreue*. For instance, Brahms and Joachim adopted the self-restraint approach to demonstrate their sincerity toward composers (Leistra-Jones, 2013).

While some musicians adopt the impersonal approach based on their beliefs in *Werktreue*, others may adopt the impersonal approach simply because they do not consider interpretation. In Reid’s (2001) categorization of students’ understanding of learning classical performance, students at the highest level actively developed interpretation and aimed “to communicate personal meaning and interpretation of the music” (p. 34). Similarly, Cantwell and Millard (1994) categorized how deeply students understood scores into five levels; students at the highest level “incorporate [ed] the literal elements of the score, but add [ed] to these an individualised interpretation” (p. 55). By contrast, students at the lower levels in both studies disregarded interpretation and expressed no personal connection to the music they played (Cantwell and Millard, 1994; Reid, 2001).

The personal approach leads to SRL, whereas the impersonal approach leads to other-regulated learning. Students with the impersonal approach in the studies above demonstrated dependence on teachers. Contrastingly, students with the personal approach explored a wide range of expression outside the lessons, seeing music-making as a process of personal development (Cantwell and Millard, 1994; Reid, 2001).

### 4.2 Explicit notation vs. implicit intention

In the professional world, just playing notes is insufficient; however, playing a note not written on a score may also be criticized (Kingsbury, 1988). Conservatory students felt anxious about changing notes (Hunter and Broad, 2017) or deviating from scores, as it implied “a violation of the composer’s wishes” (Wise et al., 2017, p. 158). Nevertheless, how rigidly professional musicians follow scores varies. Observational studies revealed that some professional musicians neglect (Kingsbury, 1988; Héroux, 2018) or change notations (Hultberg, 2002) for expressivity or practicality of performance. In contrast, other musicians adhere to markings on scores rigidly (Hultberg, 2002; Héroux, 2016). Some musicians detest using edited scores because the editors violated the composer’s original intention (Kingsbury, 1988). This explicit notation approach originated from the *Werktreue* ideology: “to be true to a work is to be true to its score” (Goehr, 1992, p. 231).

While some musicians follow explicit notations rigidly because they want to (Héroux, 2016; Payne, 2016), other musicians do so because they do not interpret the implicit intention behind the notations. This is observable in the aforementioned study by Cantwell and Millard (1994). Students who did not consider interpretation failed to draw musical meanings from notations and focused on the correct execution of the notes.

These approaches may affect exploration and self-efficacy in practice. Obedience to notations can restrict musicians’ interpretive possibilities, whereas a focus on implicit meaning allows musicians to explore wide possibilities (Reid, 2001; Hultberg, 2002; Silverman, 2008; Wise et al., 2017). In contrast, musicians placing a score superior to themselves discarded personal musical ideas if the ideas did not match with the score and exhibited a low level of confidence in their musical decisions (Hultberg, 2002; Héroux, 2016).

### 4.3 Teacher-centered vs. student-centered

Wise et al. (2017) examined how conservatory students develop interpretation in practice and found that teachers had a significant impact despite their physical absence in practice rooms. Students regarded teachers as “trustworthy authorities,” and they rarely rejected the teacher’s ideas, with one student describing it as “betraying the teacher” (p. 158). This implies that while composers’ intentions and scores are the ultimate authorities in the classical music field (Hunter and Broad, 2017), for some students, teachers are the most influential authorities, and composers and scores are seen as secondary.

The teacher-centered approach is related to the *Werktreue* ideal. Since the foundation of conservatories in the 19th century, teachers have systematically passed down the *Werktreue* ideal and “faithful” interpretations. This was negatively perceived by some musicians, including Liszt, as it fostered “a dry, pedantic, and conservative approach, hopelessly devoid of inspiration or spontaneity” (Hamilton, 2008, p. 190).

Although some musicians may take the teacher-centered approach because they value the teacher’s aesthetic and intellectual ideas, others may do so simply because they do not have their own ideas. This is shown in Reid’s (2001) study. Students who did not consider interpretation as part of musical learning relied on their teachers’ instructions. The teacher-centered approach may impair SRL, as it prevents students from exploring musical ideas that contradict teachers’ ideas in practice (Wise et al., 2017). It also makes students passive in lessons (Reid, 2001).

### 4.4 Reproductive vs. improvisatory

Hallam (1995) interviewed 22 professional musicians and found varied attitudes toward spontaneity in interpretation. Some musicians rigorously followed one interpretation in a performance that was planned and rehearsed in advance. In contrast, others left some musical choices open to maintain freshness in performance. The former approach can be categorized as reproductive, and the latter as improvisatory.

The reproductive approach is a by-product of the *Werktreue* ideal. Before the emergence of the ideology, improvisation and the improvisatory approach to the performance of pre-composed pieces were regular performance practices (Goehr, 1992; Hamilton, 2008). However, these practices gradually diminished in the 19th century, as people started to criticize improvisation as a “circus act” or “badly composed works” and altering notes in scores as violation of the composer’s intent (Goehr, 1992, pp. 233, 234).

Some musicians may adopt the reproductive approach to pursue their ideological stances, whereas others may adopt it simply because they have not grasped the musical meanings of the piece. This is implied in Hallam’s (2001) study on musicians’ metacognitive skills. She stated that musicians need to “develop accurate internal aural representations of the works” for effective learning and found that professionals had more sophisticated metacognitive skills than novices (p. 38). While the professional musicians were open to taking the improvisatory approach, none of the novice musicians considered the possibility of being spontaneous in performance. This suggests that without capturing musical characters, one cannot flexibly change musical parameters, such as tempo and dynamics, for musical expression.

The improvisatory approach contributes to effective SRL. Dolan et al. (2013, 2018) compared the improvisatory and the reproductive approaches and revealed the former immediately benefitted performers. Musicians felt that the “‘let go’ mindset” in the improvisatory approach allowed them to take risks in musical choices (Dolan et al., 2018, p. 12), and electroencephalogram (EGG) data supported that the musicians experienced a flow state. Moreover, a musicologist-researcher Dolan rated the improvisatory performances as more expressive and coherent to the score, and the audience rated the improvisatory performances higher than reproduced performances regardless of the difference in their backgrounds of musical training (Dolan et al., 2018). Similarly, classical musicians who regularly incorporate improvisatory elements found that the improvisatory approach released their music performance anxiety, as they were not restricting themselves to only what was rehearsed beforehand (Hill, 2017). In addition, students who had interpretive ideas spontaneously explored different ideas in practice (Reid, 2001).

### 4.5 Unconscious vs. conscious

In Hallam’s (1995) study, some musicians referred to interpretation as an “unconscious and intuitive process,” which will “take care of itself” (p. 120). Some musicians “did not consider interpretation at all, often, although not always, for contextual reasons” (p. 127). In contrast, some musicians developed interpretations strategically, adopting an analytic approach.

While Hallam (1995) labeled the former approach as intuitive, this approach may stem from a lack of awareness of interpretation rather than musical intuition. Ignorance of interpretation is common among students as Woody (2000) revealed that 48% of 46 music-major sophomores were unaware of expressivity until they entered high school or college. Interestingly, Hallam (1995) found that fewer musicians with the intuitive approach established personal styles in interpretation compared to musicians with the analytic approach. To differentiate it from the use of musical intuition in interpretation, we labeled the intuitive and analytic approaches as unconscious and conscious, respectively. As previously discussed, students unaware of interpretation exhibited a shallow understanding of scores, dependence on teachers, and limited exploration in practice (Cantwell and Millard, 1994; Reid, 2001; McPherson et al., 2019).

### 4.6 Separated vs. integrated

Some musicians grasp an overall musical character from sight-reading and never lose interpretive ideas even when they work on segments for technical improvement (Hallam, 1995; Chaffin et al., 2003; Holmes, 2005; Wise et al., 2017; Héroux, 2018). Other musicians neglect interpretation when they focus on technical aspects (Hallam, 1995). We labeled the former approach as an integrated approach. Musicians who adopt this approach consider interpretation regardless of whether they are sight-reading or working on sections. The latter approach was termed separated, and musicians who adopt this approach disregard interpretation when they focus on techniques. Despite how the separated approach deviated from the *Werktreue* ideology is vaguer compared to other approaches, Hunter (2005) notes that since the end of the 18th century, performance treaties started to focus exclusively on techniques, separating interpretive skills from instrumental techniques explicitly.

The integrated approach indicates the active development of interpretation, whereas the separated approach may indicate a lack of the development of interpretation. In the above-mentioned study, Hallam (1995) found that musicians who grasped the overall picture from the beginning of learning a piece attained their personal style of interpretation more than those who did not. Reid (2001) demonstrated that students who were unaware of interpretation failed to comprehend the overview of the music, perceiving music as a series of disconnected technical segments that can be worked separately. In contrast, students who aimed to communicate interpretation to audiences sought musical meaning from the sight-reading phase, and they worked on sections to integrate them into one piece (Reid, 2001).

The integrated approach benefits one in practice. Wise et al. (2017) observed a horn student continuously had interpretive ideas from the initial sight-reading and honed skills effectively to communicate the intended interpretation. This student regarded practice as a process of integrating techniques and expression. Similarly, Holmes (2005) interviewed two expert string musicians and found that their technical choices, such as fingerings and bowings, were guided by their interpretation. The musicians also avoided practicing on segments excessively to maintain the whole picture of the piece. In contrast, music students with the separated approach practiced techniques without a musical aim, resulting in ineffective practice (Reid, 2001; McPherson et al., 2019).

### 4.7 Summary

We reviewed six pairs of interpretive approaches found in music psychological research. We explored how one approach may be adopted by musicians with a lack of interpretive autonomy, resulting in other-regulated learning, whereas the other approach indicates interpretive autonomy in musicians, helping musicians initiate SRL. We categorized the former approaches as other-oriented and the latter as self-oriented interpretive approaches (Table 1). Yet how musicians’ interpretive autonomy is promoted or hindered remains unexplored. To address this issue, we will present a model of *Wekrtreue* internalization in the next section.

**Table 1:** Other- and self-oriented interpretive approaches and examples of related learning behavior.

Other-oriented interpretive approaches			Self-oriented interpretive approaches		
Approaches	Description	Examples of learning behavior	Approaches	Description	Examples of learning behavior
Impersonal	Performers restrain from imposing personal views	Failing to personally connect with music	Personal	Performers bring their personality and subjectivity into interpretations	Considering musical learning as personal development
Explicit notation	Performers follow explicit notations on a score	Failing to relate notations to musical meaning	Implicit intention	Performers neglect or change notations on a score, valuing implicit expression	Understanding musical meanings behind notations
Teacher-centered	Performers expect teachers to pass on interpretations to students	Accepting teacher’s interpretations passively	Student-centered	Performers expect students to develop their own interpretation	Evaluating teachers’ interpretation critically
Reproductive	Performers reproduce interpretations as they were rehearsed in performance	Being inflexible on stage	Improvisatory	Performers spontaneously bring new interpretations into performance	Being flexible on stage
Unconscious	Performers unconsciously develop interpretations	Lacking awareness of expressivity	Conscious	Performers consciously develop interpretations	Intentionally exploring expressivity
Separated	Performers disregard interpretations when they work on techniques	Working on segments technically without having musical aims	Integrated	Performers continually consider interpretations	Grasping an overview initially and working on techniques to express intended interpretations

## 5 The model of *Werktreue* internalization

The literature review revealed several research gaps. First, students and professional musicians have been criticized for a lack of autonomy in interpretation; however, no consensus exists on the definition of interpretive autonomy. Second, although interpretive autonomy is likely to be affected by how musicians internalize the ideology of *Werktreue*, no systematized knowledge of the mechanisms that promote or hinder musicians’ interpretive autonomy is available. Finally, although studies imply that interpretive autonomy plays a significant role in SRL and well-being, these relationships are unclear. Thus, the definition, causes, and effects of interpretive autonomy on musicians’ learning and well-being remain unclarified. To address these gaps, we propose a model of *Werktreue* internalization based on self-determination theory (Deci and Ryan, 2000).

### 5.1 Self-determination theory

Self-determination theory (SDT) has been widely applied in the fields of academics, nursing care, sports, and workplaces worldwide (Ryan and Deci, 2017). Likewise, it has been applied in the field of music (see Evans, 2015 for a conceptual overview). For instance, researchers have applied SDT to investigate music students’ motivation for study (Evans et al., 2013; Evans and Bonneville-Roussy, 2016; Miksza et al., 2021), a professional musician’s motivation for practice (López-Íñiguez and McPherson, 2020), and the degree of teachers’ autonomy-support in lessons (Kupers et al., 2013; Bonneville-Roussy et al., 2020).

SDT assumes that humans are inherently oriented toward growth. This is yet conditioned by support for basic psychological needs for autonomy, competence, and relatedness. They are universal needs defined by Ryan and Deci (2017) as “nutrients that are essential for growth, integrity, and well-being” (p. 10). People engage in growth-oriented activities “optimally only to the extent that the nutriments are immediately present or, alternatively, to the extent that the individual has sufficient inner resources to find or construct the necessary nourishment” (Deci and Ryan, 2000, p. 229).

Organismic integration theory, a sub-theory of SDT, explains the mechanism of internalization of external values. The theory posits that people are inclined to internalize social regulations into personal values to enact socially expected behavior without feeling constrained; internalization allows them to assimilate themselves into the wider society successfully.

However, optimal internalization requires the fulfillment of three basic psychological needs. The need for competence is fulfilled when an individual perceives “the ability to understand or grasp the meaning or rationale behind the regulation and an ability to enact it” (Deci and Ryan, 2000, p. 238). The need for relatedness is satisfied through “feelings of relatedness to socializing others” (p. 238). Finally, a complete internalization requires support for the need for autonomy to “freely process and endorse transmitted values and regulations (and to modify or transform them when necessary)” (p. 238).

Organismic integration theory identifies four types of regulations, which are categorized into controlled and autonomous regulations depending on the degree of autonomy. Controlled regulations are caused when people’s needs are thwarted, for example, in excessively controlling, overchallenging, and rejecting environments. In controlled regulations, people are controlled by external or internal contingencies, such as fame and a sense of guilt. Thus, their behavior is not self-determined, and “regulations and values may either remain external or be only partially internalized to form introjects or unintegrated identifications” (Deci and Ryan, 2000, p. 236). Controlled regulations lead to poor performance and ill-being since the basic psychological needs required for optimal well-being are thwarted. Therefore, controlled regulations thwart their psychological needs further, trapping them in a vicious circle.

Conversely, autonomous regulations are facilitated when people’s needs are satisfied in the process of internalization. In autonomous regulations, “people will identify with the importance of social regulations, assimilate them into their integrated sense of self, and thus fully accept them as their own” (Deci and Ryan, 2000, p. 236). Since the value is integrated into personal values and identity, the behavior is enacted autonomously. Autonomous regulations then lead to a high quality of performance and enhanced well-being (Deci and Ryan, 2000). This then satisfies the needs, promoting autonomous regulations further. In the subsequent model, we adopted the classification of controlled and autonomous to reflect different internalizations of the *Werktreue* ideal among classical musicians.

### 5.2 The model of *Werktreue* internalization

#### 5.2.1 Definition of interpretive autonomy

According to Deci and Ryan (2000), “basic needs play an essential role in cultural transmission, helping to account for how memes are assimilated and maintained in and across diverse human groups” (p. 230). In classical music, the ideology of *Werktreue* is a value that has been transmitted since the end of the 18th century. Based on SDT, a model of *Werktreue* internalization categorizes two qualitatively different internalizations of the *Werktreue* ideal: controlled and autonomous. In controlled internalization, musicians’ interpretive autonomy is hindered, whereas in autonomous internalization, musicians’ interpretive autonomy is promoted.

When the psychological needs are thwarted in interpretation, the *Werktreue* ideology remains external to the self. In interpretation, musicians are controlled by a sense of incompetence, pressure, and fear of rejection. Thus, their self-esteem is contingent on how others evaluate their interpretation, and they are highly self-critical of their interpretation. This represents the controlled *Werktreue* internalization that hinders musicians’ interpretive autonomy.

**Figure 1:**
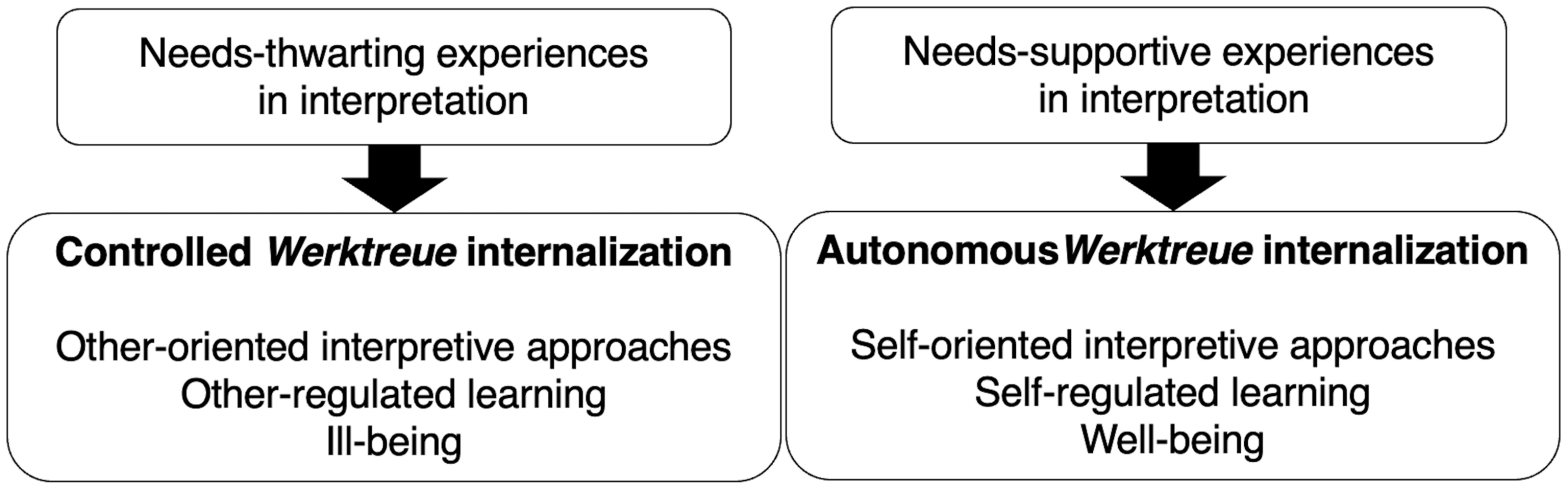
The model of *Werktreue* internalization.

In contrast, when the psychological needs are fulfilled in interpretation, the *Werktreue* ideology is fully internalized and integrated with the self. In contrast to controlled interpreters, musicians are freed from concerns of incompetence, pressure, and rejection, and they make interpretive choices based on personal interests, feelings, and intellectual curiosity. This represents the autonomous *Werktreue* internalization that promotes musicians’ interpretive autonomy (see Figure 1).

The model of *Werktreue* internalization does not define the *Werktreue* ideology or interpretation. Instead, the model identifies interpretive autonomy based on whether the ideology is alienated from or integrated with a musician’s self regardless of the musician’s definition of faithful interpretation. Performers with the autonomous *Werktreue* internalization are self-determined, and their faithful interpretation is connected with the true self. Therefore, they pursue their own ideological stance freely based on their interests and personalities. In contrast, performers with controlled internalization are ego-involved, and they are controlled by external or internalized contingent rewards and punishments when they interpret a piece. Therefore, they pursue objective faithfulness or their subjectivity out of anxiety.

By defining interpretive autonomy without defining the *Werktreue* ideal, the proposed model solves a dualism seen in previous studies. Researchers have shown categorizations based on whether musicians prioritized individual freedom or the composer’s intention over the other (Lindström et al., 2003; Laukka, 2004; Silverman, 2007; Héroux, 2018). Although this categorization is useful for differentiating varied definitions and beliefs, it does not help us identify interpretive autonomy as we discussed at the beginning of the article. Rather than pitting one against the other, the model of *Werktreue* internalization is built on an assumption that musicians modify or change the definition of the ideology throughout their lives as they interact with social environments and pass through different stages of their personal development.

#### 5.2.2 Factors that promote or hinder interpretive autonomy

The model explains the mechanisms that promote or hinder interpretive autonomy. If musicians understand the rationale behind the ideology (the need for competence), sense the freedom to modify the ideology (the need for autonomy), and feel connected with others through the ideology (the need for relatedness), their psychological needs are fulfilled. This facilitates the autonomous *Werktreue* internalization, and their interpretive autonomy is promoted. On the other hand, if musicians do not grasp the meaning of the ideology (the need for competence), perceive no freedom to modify the ideology (the need for autonomy), and feel isolated by the ideology (the need for relatedness), their needs are thwarted. This results in the controlled *Werktreue* internalization, and their interpretive autonomy is hindered.

Factors that are found to promote or hinder students’ interpretive autonomy in lessons, practice, and performance are reviewed below. The model accounts for the psychological mechanisms of how the factors promote or hinder students’ interpretive autonomy.

##### 5.2.2.1 Needs-thwarting lessons

Karlsson and Juslin (2008) analyzed eight hours of video recordings of private lessons involving 12 students and five teachers and found that the teachers’ talk dominated lessons with little demonstration of the instrument. The instrumental teachers gave technical instructions to reproduce notations and rarely mentioned interpretation or expression. Students’ playing was interrupted constantly by outcome feedback, such as “good” or “bad,” without any further explanation. Another study found that a conservatory teacher made interpretive decisions based on how “everyone” plays in lesson (Burwell, 2021, p. 470). Burwell (2021) cautioned that this reinforced social norms in interpretation and the authority of the teacher who knew the norms. In other cases, teachers ignored (Rostvall and West, 2003) or explicitly rejected students’ original interpretive ideas (Persson, 1996; Silverman, 2008; Wagner, 2015).

When interpretation or expression is addressed, instrumental teachers employ strategies such as modeling, use of metaphors, and provoking felt emotions (Woody, 2000; Lindström et al., 2003; Laukka, 2004). While these strategies can be effective, they also have some flaws. Modeling may lead students to merely copy (Laukka, 2004). Other verbal instructions can be ambiguous, leaving students confused and frustrated (Woody, 2000; Schippers, 2006; Karlsson and Juslin, 2008).

If students perceive that they have no interpretive choices, the need for autonomy is thwarted. If students feel that their original ideas are rejected by teachers, or students cannot understand teachers’ instructions, the need for competence is thwarted. These needs-thwarting experiences lead students to be internally controlled in interpretation.

##### 5.2.2.2 Needs-thwarting practice

At the beginning of learning, some parents accompany their children in practice. While appropriate parental support promotes children’s musical growth (Davidson et al., 1996; McPherson and Davidson, 2002), excessive parental involvement may hinder children’s interpretive autonomy. Wagner (2015) interviewed parents of students in soloist violin classes and revealed that the parents attended lessons, took notes or videos, and assisted their children in practice. Some children were home-schooled, and their practice was supervised by their parents who voluntarily became their teachers’ assistants. Some of the children then tried to avoid practicing by damaging their violins or leaving the instruments in school. Similarly, investigating 337 teacher-pupil-parent triads, Creech (2009) categorized interaction styles into six types; in one of the types, a “solo leader” type, a dominative teacher expected parents to assist their children’s practice to meet the teacher’s expectations. Although parents of this type gave high behavioral, cognitive, and personal support, the children rated lower scores in enjoyment of music, personal satisfaction, motivation, self-efficacy, and self-esteem than children whose parents actively negotiated with the teacher regarding the expectations, not to overtire the child (Creech, 2009, 2010).

If practice is supervised by parents who insist on children following teachers’ instructions, children have no space to explore interpretive possibilities. This thwarts the need for autonomy as they cannot choose how to play a piece.

##### 5.2.2.3 Needs-thwarting performance

Students often perform in exams, competitions, and auditions, in which their performances are evaluated normatively. In particular, competitions are greatly valued, as they may boost students’ careers (McCormick, 2015). To win in a competition, however, students need to appeal to judges by demonstrating their originality *within* accepted performance styles. McCormick (2015) investigated international competitions through observation and interviews and demonstrated that although juries emphasized the significance of individuality in interviews, they also detested competitors whose interpretations deviated from stylistic conventions or scores, regarding them as uneducated or disrespectful musicians. In addition, judges differed in their understanding of what authentic interpretation is. Therefore, unconventional interpretation often resulted in split juries, and those musicians who conveyed highly original interpretations were eliminated early due to the voting or scoring system.

Pressures to conform to standardized interpretation may thwart the need for autonomy. Moreover, rejection from juries thwarts the need for competence and relatedness, making musicians psychologically controlled interpreters.

##### 5.2.2.4 Needs-supportive lessons, practice, and performance

Several factors are found to promote interpretive autonomy. Open questions and discussions of musical characters are suggested to be effective for conservatory students (Hultberg, 2002; Young et al., 2003; Burwell, 2005; Nerland, 2007). Notably, this was effective for pre-teens and teenagers (Meissner, 2017; Meissner and Timmers, 2019, 2020; Trapkus, 2020; Meissner et al., 2021). After being aware of musical expression, children showed intrinsic motivation to learn to “make pieces ‘their own,’” even when the pieces were technically challenging (Meissner and Timmers, 2020, p. 14). If students perceive that they have capability and choices in musical interpretation through open-ended questions and discussions, they autonomously internalize the *Werktreue* ideal, and their interpretive autonomy is promoted.

Exploration in practice may also fulfill the needs. McPherson and McCormick (1999) found that children who engaged in both formal and informal practice reported higher enjoyment than other children. In addition, informal practice was engaged more by high-achieving students than low-achieving students (Sloboda et al., 1996). Playful informal practice fulfills the need for autonomy, as students can freely try different interpretive ideas without pressure. Furthermore, improvisation (Hill, 2017) and listening to different interpretations expose students to diverse interpretive choices (Silverman, 2008; Volioti and Williamon, 2017, 2021), satisfying the need for autonomy. This suggests that providing a secure place where students can explore interpretation by freely manipulating performance cues, such as dynamics and tempo, is effective in supporting the development of interpretive autonomy.

Finally, performance experiences in non-traditional venues, such as hospitals and nursing homes, with no contingent rewards and punishments for musical decisions may support the psychological needs. Nine conservatory students participated in a 10-week program where they helped group music-making at nursing homes in Switzerland by playing and singing a wide variety of music, and this had a positive impact on the students (Paolantonio et al., 2022). The students received immediate reactions from the participants as they played music, and the students were moved by how intensely the older people appreciated music. Through the program, some students realized that they were trapped in a narrow mindset and standards, and they reconsidered the personal meaning of making music (Paolantonio et al., 2022). Also, Perkins (2013) found that a student appreciated performance opportunities outside a conservatory as her performance opportunities were limited within the conservatory due to the rigid hierarchies. In those opportunities outside the school, she could “make mistakes and learn,” unlike performances in school (p. 206). Considering students are pressured to make appropriate interpretative decisions within conservatories (Hunter and Broad, 2017) and competitions (McCormick, 2015), these opportunities outside the classical music field offer a valuable space for students. Without being pressured to succeed, students can explore interpretive possibilities through interactions with a wider audience. If students perceive that they can choose and convey their personal interpretation and connect with others through performance, these performance experiences promote students’ interpretive autonomy.

#### 5.2.3 The effects of internalization types

In addition, the proposed model accounts for the effects of interpretive autonomy on musicians’ learning and well-being.

##### 5.2.3.1 Interpretive approaches

In the previous section, we introduced six pairs of different interpretive approaches and classified them into two categories: other-oriented and self-oriented. Other-oriented interpretive approaches deviated from the *Werktreue* ideal but may be adopted by musicians with a lack of interpretive autonomy. Conversely, self-oriented interpretive approaches are taken by musicians with interpretive autonomy.

Other-oriented interpretive approaches are likely to be adopted by musicians with the controlled *Werktreue* internalization. This is because other-oriented interpretive approaches do not require competence, autonomy, and relatedness to be employed. For instance, musicians who want to avoid punishment obey explicit notation or their teachers’ interpretation to avoid being accused of unfaithfulness. In contrast, self-oriented interpretive approaches are adopted by musicians with the autonomous *Werktreue* internalization, as these approaches require a sense of autonomy, competence, and relatedness to be employed. For instance, the improvisatory approach, in which musicians make musical decisions spontaneously, requires autonomy and competence, as musicians need to make musical choices instantly.

Musicians with the autonomous *Werktreue* internalization can also employ other-oriented interpretive approaches purposefully to pursue their *Werktreue* ideals. For instance, in Payne’s (2016) case study, a musician explicitly stated that he intended to express the work itself, not himself. He adhered to the score strictly but also developed a highly individualized interpretation. This “paradox” (p. 339) is dissolved if he has internalized the ideology autonomously and freely chosen to follow the text to reveal the composer’s intention. These musicians, wishing to let the music speak for itself, would employ both self-oriented and other-oriented interpretive approaches.

Musicians with the controlled *Werktreue* internalization face difficulty in employing self-oriented interpretive approaches, as these approaches require a sense of competence, autonomy, and relatedness in interpretation to be employed. For example, a student anxious about the appropriateness of interpretation would depend on teachers’ interpretive ideas and might not explore fresh ideas on stage, taking the teacher-centered and the reproductive approaches. Therefore, musicians with controlled internalization employ only other-oriented interpretive approaches.

##### 5.2.3.2 Self-regulated learning and well-being

In the first section, we discussed how interpretative autonomy contributes to SRL, and how SRL leads to enhanced well-being. Conversely, a lack of interpretive autonomy results in other-regulated learning behavior and ill-being. As students with more autonomous regulations demonstrate better learning behavior and well-being (Deci and Ryan, 2000), the model of *Werktreue* internalization integrates the effects of internalizations on SRL and well-being compatibly.

##### 5.2.3.3 Musical identity

SDT considers the effects of types of internalizations on identity. With autonomous regulations, the external value is fully integrated with one’s sense of self, whereas with controlled regulations, the value remains alienated from the self; thus, the identity is disintegrated in the activity (Deci and Ryan, 2000). This implies that musicians with autonomous internalization, who perceive no constraints in expressing original interpretation, acquire an integrated identity as a classical musician. In contrast, musicians with controlled internalization, who feel restricted in expressing personal interpretations, face difficulty embracing an identity as a classical musician.

#### 5.2.4 Other key points

##### 5.2.4.1 Interpretive autonomy as a state

SDT posits that individuals can “internalize a new behavioral regulation at any point … depending on both prior experiences and current situational factors” (Ryan and Deci, 2000, p. 73). Thus, interpretative autonomy can be considered context-specific; while earlier experience does affect later development, types of internalizations shift as the degree of needs satisfaction changes. This means that interpretive autonomy hindered by needs-thwarting environments can be promoted if musicians move to more needs-supportive environments. Silverman (2008) reported a case study of a Russian pianist who went through needs-thwarting experiences but subsequently regained interpretive autonomy. The pianist studied with an authoritarian teacher who allowed no interpretive freedom. The teacher forced him to reproduce the teacher’s own interpretations and techniques:

“[The teacher] damaged, for me, all composers I played. He gave me ‘schooling’—a discipline of ear and fingers, no question about that. But speaking stylistically, about being authentic, he completely distorted everything that my intuition, if left alone, would have understood. If he did not intervene in such a way, I would have instinctively found my way to different styles.” (Silverman, 2008, p. 263).

In the face of adversity, the pianist avoided needs-thwarting experiences by refusing to enter competitions and choosing to perform pieces that the teacher could not teach. This allowed him to focus on the development of personal interpretation. The pianist demonstrated high interpretive autonomy, adopting self-oriented interpretive approaches introduced in the previous section. However, the pianist required more than a decade to gain confidence and find his artistic voice in interpretation.

##### 5.2.4.2 Types of internalizations and teaching style

Persson (1996) observed a teacher rejecting students’ original musical ideas in lessons and concluded that the authoritarian teacher aimed to convey “artistic life” to his students, in which “commitment is crucial—commitment to others’ expectations, prompted by established and inflexible traditions, rather than one’s own artistic convictions” (p. 41). The teacher believed that no opportunities existed for musicians to develop personal interpretations in the professional world and felt responsible to teach that in his lessons. Similarly, Hultberg (2002) suggested that teaching styles are affected by teachers’ own approaches to interpretation. Teachers adhering to explicit markings do not allow students to develop individual interpretations that deviate from a score. In contrast, teachers who value implicit meanings and take the personal approach to interpretation welcome students’ original ideas, and they engage in discussion with students as “co-creative interpreters in a communication with the composer” (p. 195). This implies that teachers internalize the *Werktreue* ideal differently depending on their psychological experiences as students and professional musicians in the classical music field. This affects their teaching styles, possibly transmitting their types of internalizations to their students.

##### 5.2.4.3 The dualistic model of passion

Although we adopted SDT, another similar theory exits, that is, the dualistic model of passion (Vallerand et al., 2003; Vallerand, 2010). The dualistic model of passion is based on SDT and considers only internalizations of activities that are related to one’s identity. An autonomous internalization of the activity leads to harmonious passion, whereas a controlled internalization of the activity leads to obsessive passion (Vallerand et al., 2003; Vallerand, 2010). Studies among expert musicians confirmed that harmonious passion was associated with mastery goals and well-being, whereas obsessive passion was correlated with both mastery goals and performance goals and negatively correlated with well-being (Bonneville-Roussy et al., 2011; Bonneville-Roussy and Vallerand, 2020).

Since musicians’ identity is often connected with music, the dualistic model of passion is insightful. However, we applied SDT to include learners whose identity is not related to music, such as children and beginners. McPherson (2005) highlights the significance of having interpretive ideas from the beginning of instrumental learning; the study revealed that mental strategies that included having musical ideas predicted children’s achievements in sight-reading, playing from memory, and playing by ear in the first three years of learning. However, the ability to perform rehearsed music was explained little by the use of mental strategies (McPherson, 2005). This implies that a lack of interpretive autonomy may be unnoticed as long as students can reproduce what was rehearsed beforehand especially in the early years of learning.

## 6 Discussion and conclusion

The model of *Werktreue* internalization identifies the definition, causes, and effects of interpretive autonomy. Musicians who perceive that they are capable of interpretation, have musical choices, and are connected with others through faithful interpretation, autonomously internalize the ideology of *Werktreue.* In autonomous internalization, musicians are fully self-determined in interpretation, thus they employ self-oriented interpretive approaches. This supports SRL and positive well-being, and their musical identity is integrated. Conversely, musicians who perceive that they are incapable, have no musical choices, and are isolated through faithful interpretation, internalize the ideology in a controlled form. In controlled internalization, musicians’ self-worth is contingent on interpretation; thus, they use other-oriented interpretive approaches. This results in other-regulated learning and poor well-being, and their musical identity is disintegrated.

The model of *Werktreue* internalization contributes to both academia and educational practice by addressing three knowledge gaps. First, it defines interpretive autonomy based on types of internalizations without defining interpretation or the *Werktreue* ideal. Second, it demonstrates the importance of interpretive autonomy in SRL and well-being explicitly. Third, the model provides plausible explanations of the mechanisms that facilitate or inhibit interpretive autonomy in learning.

Based on the model, researchers and educators can assess musicians’ degree of interpretive autonomy by observing their interpretive approaches, learning behavior, and well-being. Moreover, they can promote interpretive autonomy by fulfilling the basic psychological needs in interpretation. They could empower learners with interpretive knowledge and skills, provide musical choices, and offer performance opportunities where learners can connect with the audience through personal interpretation. For specific teaching strategies to promote expressivity or interpretation in lessons, frameworks (Hultberg, 2002; Meissner, 2021) and a case study on successful teachers (Nerland, 2007) are useful. The model of *Werktreue* internalization aligns with these studies, accounting for the effectiveness of the teaching methods from the psychological perspective of learners. Yet a limitation of this study is that the model is built based on the literature. Further empirical studies are warranted to test the plausibility of the model.

We noted that authoritarian teaching, excessive parental control in practice, and competitions have risks of hindering interpretive autonomy. However, if they support students’ basic psychological needs in interpretation, they may be effective for students’ growth. It is important to keep in mind that needs fulfillment is subjective, and learning experiences, such as competitions or authoritarian teaching, may be perceived as needs-thwarting for one while it may be perceived as needs-supportive for another student.

Nevertheless, this implies high risks of interpretive autonomy being impeded for musicians who are professionally educated from the early years of life. Students aiming to become concert soloists often go through authoritarian teaching, excessive parental control in practice, and rejections in competitions (Wagner, 2015). If these learning experiences thwart their basic psychological needs, their interpretive autonomy is hindered, potentially damaging their long-term artistic development in the early years of their music study. Even after they acquired high techniques, these musicians would be regulated by normative “authentic” interpretations. Being anxious about their interpretations, they would display other-regulated learning behavior, such as vulnerability against feedback. This endangers their physical and psychological health. Nevertheless, because performing is deeply entrenched in the identity, they would persist in performance careers while being psychologically controlled in expressing interpretation.

Interpretation is at the heart of classical musicians’ learning and well-being. It is a process in which musicians cultivate intellect and empathy to find their own voice in a piece that they want to share with others. This study does not reject the *Werktreue* ideal or normative interpretations; the *Werktreue* ideal has encouraged musicians to make the most of music, and normative interpretations have become a standard because many people are moved and convinced by the interpretation. However, depending on how music students perceive and internalize the *Werktreue* ideal in learning, the ideology may become a burden, making musicians restrict their expressive freedom and strive for conformity. To support musicians’ life-long musical and human development, it is imperative to consider how we can provide students with psychological support so that they can explore their artistic voice from the beginning of their study.

## Data availability statement

The original contributions presented in the study are included in the article/supplementary material, further inquiries can be directed to the corresponding author.

## Author contributions

MF: Conceptualization, Writing – original draft, Writing – review & editing. YU: Writing – review & editing, Funding acquisition, Supervision.

## Footnotes

^1^We use *other-regulated learning* to describe a learning style regulated by others to contrast it with self-regulated learning.

^2^It should be noted that Chen (2023) reported descriptive statistics.
